# Aerobic scope and temperature preference in yellow tang (*Zebrasoma flavescens*) at current and elevated sea temperatures

**DOI:** 10.1007/s00360-025-01627-y

**Published:** 2025-08-26

**Authors:** Elsa S. van Hall, Keith E. Korsmeyer

**Affiliations:** Department of Marine Science, Hawaiʻi Pacific University, 1 Aloha Tower Drive, Honolulu, HI 96813 USA

**Keywords:** Acclimation, Climate change, Energetics, Hawaii, Metabolism, Temperature

## Abstract

Climate change is predicted to continue elevating regional sea surface temperatures (SST) and increase the frequency and severity of localized heating events, phenomena which may threaten the biodiversity, integrity, and function of tropical coral reef ecosystems. The primary objective of this study was to determine physiological and behavioral responses to elevated SST in a Hawaiian surgeonfish, the yellow tang, *Zebrasoma flavescens.* We assessed standard metabolic rate (SMR), maximum metabolic rate (MMR), aerobic scope (AS), and swimming performance, as well as temperature preference (T_pref_) in this ecologically and economically important coral reef fish. The *Z. flavescens* were acclimated to either the current maximum monthly summer SST around Oʻahu, 27 °C, or an elevated SST, 31 °C. Acclimation temperature had no significant effect on SMR, MMR, AS, or swimming performance. Temperature preference was tested over a 24-hour period in an annular preference chamber with a gradient ranging from 24 to 34 °C. Our study found that *Z. flavescens* in both acclimation temperatures had a similar T_pref_ (median) of 27 °C with first and third quartiles of 25.7 to 29 °C. Analysis of relative use of available temperatures (compositional analysis) indicated a preference for the lowest available temperatures of 24 to 26 °C in both acclimation groups. These findings indicate that *Z. flavescens* can completely compensate AS and swimming ability to the elevated SST conditions, although T_pref_ remains near or below the current summer SST, suggesting other factors explain behavioral temperature preference.

**Supplementary Information:**

The online version contains supplementary material available at 10.1007/s00360-025-01627-y.

## Introduction

Contemporary marine species are experiencing unprecedented temperature increases in coral reef ecosystems, with current rates of change far exceeding those seen over the past 800,000 years, driven by anthropogenic greenhouse gas emissions (IPCC et al. 2013, 2014; Nowicki et al. 2012; Pandolfi et al. 2011). Since the 1970 s, warming oceans have prompted some marine fishes to shift to cooler, higher latitudes, a movement projected to continue as temperatures rise (Dahlgren and Eggleston 2000; Hoegh-Guldberg et al. 2014; Perry et al. 2005; Pörtner 2001). Due to their narrow thermal tolerance, tropical species are highly susceptible to increasing sea surface temperatures (SST; Habary et al. 2017). Many tropical fishes are expected to be near their thermal maxima already, therefore it is important to understand how further temperature increases could impact their physiological performance (Habary et al. 2017; Johansen and Jones 2011; Messmer et al. 2017; Rummer et al. 2014; Velasco-Blanco et al. 2019).

Aerobic scope (AS) serves as a key indicator of a species’ tolerance to environmental changes, providing insights into thermal niches and potential responses to rising ocean temperatures or marine heatwaves (Johansen and Jones 2011; Pörtner and Knust 2007). A fish’s ability to uptake oxygen in support of aerobic metabolism is limited by lower and upper thresholds. The lower threshold, or standard metabolic rate (SMR), is the subsistence level in a stress-free environment and the upper threshold, or maximum metabolic rate (MMR), typically occurs when the fish is swimming at a maximum prolonged swimming speed. The difference between these thresholds, the absolute aerobic scope, represents the available aerobic capacity for fitness related activities (e.g., locomotion, reproduction, growth, foraging; Clark et al. 2013; Fry 1947; Pörtner and Knust 2007; Roche et al. 2013). For example, swimming performance is vital for survival, aiding in predator evasion and prey capture and is temperature-dependent with optimal temperatures allowing maximum performance and declines occurring as temperatures deviate from this optimum (Johansen and Jones 2011; Jutfelt 2020; Plaut 2001).

The ‘oxygen- and capacity-limited thermal tolerance’ (OCLTT) hypothesis suggests that as body temperatures increase beyond an optimum in ectotherms, the oxygen supply decreases in relation to oxygen demand in the tissues for ATP synthesis, leading to a smaller AS and diminished performance (Lefevre et al. 2021; Pörtner and Knust 2007; Pörtner 2017). The OCLTT hypothesis has gained popularity to link performance changes that are influenced by temperature to ecological and biogeographical changes (Pörtner 2001, 2021; Pörtner et al. 2017; Schulte 2015). However, several studies found that AS was not reduced at higher temperatures and did not correlate with measures of fitness or performance in some marine species (Gräns et al. 2014; Kirby et al. 2020; Raby et al. 2016); therefore, more evidence is needed to corroborate the hypothesis.

Temperature preference is a crucial aspect of ecology that influences a species’ distribution, behavior, fitness, and resource availability. As climate change alters environmental temperatures, understanding how species select their thermal environments becomes increasingly important. Temperature preference can impact physiological processes such as metabolism, growth, and reproduction, thereby affecting population dynamics and community structure. Some species can behaviorally regulate their body temperature within a range of temperatures, allowing them to maintain preferred values (Christensen et al. 2021). This temperature preference has been used as a proxy for optimal temperature, where physiological and biochemical processes may be maximized, resulting in optimal fitness, and may indicate how the species will respond to environmental temperature change (Christensen et al. 2021; Habary et al. 2017; Jobling 1981; Macnaughton et al. 2018; Magnuson et al. 1979). However, temperature preference is not always equivalent to optimal temperature, as aerobic scope can continue to increase until temperatures approach lethal levels (Clark et al. 2013).

The objectives of this study were to determine the physiological and behavioral responses of yellow tang, *Zebrasoma flavescens* (Bennett 1828) to rising sea surface temperatures (SST). This species was selected due to its prevalence as an algae grazer on Hawaiian coral reefs, its relatively restricted range in the North Pacific tropical and subtropical ocean, and its importance in aquaculture and the aquarium trade (Bernardi et al. 2018; Callan et al. 2018; Claisse et al. 2009; Randall 2007; Walsh et al. 2004). *Zebrasoma flavescens* grazing patterns help regulate algae growth, preventing fast-growing algae from outcompeting slow-growing corals for space and sunlight (Tissot and Hallacher 2003). Juveniles inhabit mid-depth (5–12 m) branching coral habitat and utilize coral structures for predator protection (Ortiz and Tissot 2008). As they mature, *Z. flavescens* transition to shallow (< 5 m) reef flats during the day to forage and move to deeper water at night to shelter from predators, although the temperature changes are minimal as these movements are within the upper mixed-layer (< 30 m) (Claisse et al. 2011; Ortiz and Tissot 2008).

We investigated the aerobic metabolic scope and swimming performance of *Z. flavescens* acclimated to either the current average monthly maximum SST around Oʻahu, Hawaiʻi (27  °C), or an elevated end-of-century SST (31  °C), which exceeds their typical range (24.9–28.9 °C, Myers 1991; IPCC 2014). Focusing on acclimation responses, rather than short-term, acute effects, aligns with the timescales of projected SST increases (Seebacher et al. 2014). Additionally, we assessed temperature preference in relation to acclimation temperature (27–31 °C) using an annular preference chamber. We hypothesized that their performance, as measured by aerobic scope, would be optimized at the contemporary temperature (27 °C) and reduced at the elevated temperature (31 °C). A smaller aerobic scope at elevated temperatures would reduce oxygen supply capacity for fitness-related activities and, therefore, we predicted reduced swimming performance in the elevated acclimation temperature (31  °C; Clark et al. 2013; Johansen and Jones 2011; Pörtner and Knust 2007). Our second hypothesis was that *Z. flavescens* would prefer temperatures near their current habitat temperature (27 °C), where the species has adapted. This study provides insight into the physiological and behavioral responses of an economically and ecologically important Hawaiian surgeonfish to rising SST, supporting conservation efforts for reef species.

## Materials and methods

### Animal collection and handling

*Zebrasoma flavescens* (*n* = 19, total length = 8.0 to 11.4 cm, mass = 11.8 to 32.4 g) were purchased through a wholesale collector, Hawaiian Sea Life Inc., that caught them in near-shore waters off the island of O’ahu, Hawaiʻi, and temporarily held them in a recirculating seawater system. The fish were transported to and held at the Makapuʻu Campus of Hawaiʻi Pacific University (Waimanalo, HI) in aerated, flow-through seawater indoor tanks (Salinity 30–32 PSU, 12 h:12 h light-dark photoperiod). The fish were provided with strips of dried seaweed available for grazing throughout the day (~ 400 cm^2^ total daily per tank, tied to pieces of coral rubble), and fed to satiation once a day with commercial marine fish pellets (~ 4 g per tank).

### Temperature acclimation

The *Z. flavescens* were randomly divided into two groups and acclimated to either 27 °C (*n* = 10) or 31 °C (*n* = 9) for at least four weeks before being used in experiments. The fish were housed with their respective treatment groups in circular fiberglass tanks, with the 31 °C acclimation group in a 600 L tank (156 cm diameter, 32 cm depth) and the 27 °C acclimation group in an 800 L tank (152 cm diameter, 44 cm depth). The tanks were identical in all other conditions. For the 31 °C acclimation group, the temperature was increased by 0.5 °C every two days from ambient seawater temperature (~ 27 °C) until the desired temperature (31 °C) was reached, over 17 days. The fish were then maintained at 31 °C for an additional four weeks before the experiments began. Water temperatures were regulated (± 0.2 °C) using a Digital Temperature Controller (Bayite, BTC201, Shenzhen, China), to activate submersible heaters or a pump to circulate tank water through a stainless-steel coil submerged in a chilled water bath. Prior to each experimental test, the fish were fasted for at least 24 h to minimize postprandial metabolism. To isolate individual fish for fasting or holding prior to the respirometry and temperature preference experiments, the fish was moved into a floating mesh fish basket (48 cm diameter) inside the respective acclimation tank. Each fish was tested individually, first in the swimming respirometer and then, 48 to 96 h later, in the annular temperature preference chamber.

### Swimming respirometry

Automated, intermittent-flow swimming respirometry was used to measure the oxygen consumption rate for each *Z. flavescens* when at rest (standard metabolic rate, SMR) and during maximum prolonged swimming (maximum metabolic rate, MMR; Roche et al. 2013; Steffensen et al. 1984; Svendsen et al. 2016). The acrylic, 6.7 L swimming respirometer was inside of an external water tank (52.4 L) that drained to an aerated, temperature-controlled sump (52.0 L), from which water was pumped through a UV sterilizer back into the external tank (Fig. S1; Schakmann et al. 2020). Water from the external tank could be flushed through the respirometer by activating a submerged pump. The respirometer was tested for leaks by pinching the flushing tube while the water level was elevated in the overflow tube. If the water level remained stable, the chamber was considered leak-free. Temperatures in the swimming respirometer were maintained at the fish acclimation temperature (± 0.1 °C). A variable-speed motor connected to the propellers drove the water first through a flow straightener and then the swimming section (20.3 cm long, 8.9 cm wide, 8.9 cm deep) in which the fish was placed. An optical dissolved oxygen (DO) probe with temperature sensor and a conductivity probe inserted through the lid of the swimming respirometer were used to continuously record oxygen content (every 5 s) with automatic correction for salinity and temperature (Multi 3430 multimeter, Wissenschaftlich Technische Werkstätten [WTW], Weilheim, Germany). The DO probe was calibrated to 100% air saturation before each use, following the manufacturer’s recommendations. The data from the multimeter were acquired and analyzed with custom respirometry software (LabVIEW 2017; National Instruments, Austin, TX) that also controlled the intermittent flushing of the respirometer with a computer-actuated relay (USB-SwitchC, Cleware GMBH, Hollingstedt, Germany).

The afternoon before the day of the swim test, the fish’s mass, total length, depth, and width were quickly measured prior to placement in the swimming section of the respirometer to allow calculation of relative swimming speeds (body lengths per second, BL s^−1^) and correct for solid-blocking effects (Bell and Terhune 1970; Korsmeyer et al. 2002). The water speed was kept low at ~ 5 cm s^−1^ (~ 0.5 BL s^−1^), which was enough for water mixing, but did not induce swimming. This flow was to allow recovery from handling and then overnight (19:00–05:30) measurements of oxygen consumption rates (*Ṁ*_O2_) for determination of SMR (Svendsen et al. 2016). For overnight measurements, each respirometry cycle was automated at 12 min consisting of a 4 min flushing period with air-equilibrated water from the external tank to return oxygen levels to near air-saturation (> 95%), a 1 min closed mixing period for the observed steady draw down in dissolved oxygen to begin, and a 7 min closed measuring period for a sufficient drop in oxygen content to be recorded for determination of resting metabolic rates (Svendsen et al. 2016).

For swimming *Ṁ*_O2_ measurements the next morning, the measuring period regulated by the LabVIEW software was reduced to five minutes because the higher metabolic rates during swimming allowed a shorter period for observing a consistent decline in oxygen content, resulting in 10 min intermittent respirometry cycles (4 min flushing period, 1 min closed mixing period, and a 5 min closed measuring period). The water speed was gradually increased over 2 min to 3.0 BL s^−1^ and *Ṁ*_O2_ was recorded over three 10 min cycles, or 30 min at that speed. The water speed was then increased by 0.5 BL s^−1^ every 30 min until the fish was not able to maintain position in the swimming section and fell back against the flow straighteners twice. During each measurement period, the fish was video recorded (GoPro, Hero 3+) for one minute to allow determination of gait transition speeds. Following the swimming trial the fish was returned to the mesh holding basket in its acclimation tank and was used in a temperature preference trial within 96 h. Background *Ṁ*_O2_ was measured by conducting at least three 12 min cycles in the respirometer preceding and immediately following the trial. Following each experiment, the entire system was disinfected with bleach and rinsed with fresh water.

### Temperature preference

**Fig. 1:**
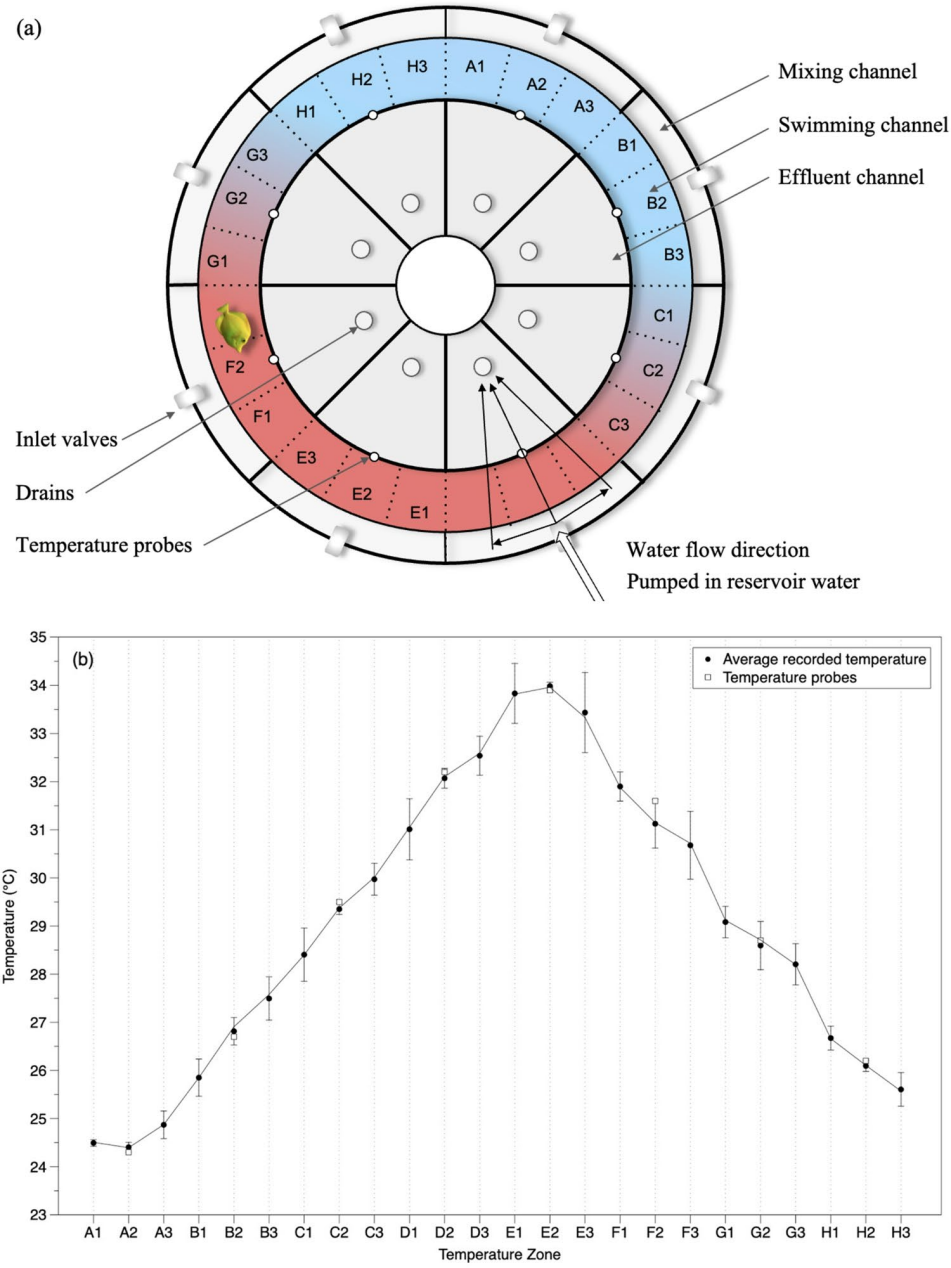
Annular temperature preference chamber. **a** Water from five reservoirs was recirculated into the temperature preference chamber through the inlet valves to one of eight zones (i.e., A2, B2, C2,…H2) to create the temperature gradient in the swimming channel. **b** Example of average water temperatures with standard deviation in 24 temperature zones (A1-H3) measured post experiment. Temperatures were measured by the outer wall, middle and inner wall of the swimming channel at three depths in the water column: top of the water column (1 cm), middle of the water column (5 cm), and bottom (10 cm). In addition, water temperatures were continuously measured during the experiment with a temperature probe that was affixed to the middle of the inner wall of the swim channel in each temperature zone (open squares)

The fish temperature preference (T_pref_) was tested using an annular temperature preference chamber similar to the design by Myrick et al. (2004) with modifications for water recirculation as in Schram et al. (2013; Fig. 1, Fig. S2). The chamber (61.6 L) was constructed from PVC and consisted of four concentric cylinders to create an outer mixing channel (outer wall radius of 45 cm) that received temperature-regulated seawater, a swimming channel (outer radius of 40 cm) where the fish was placed and inner effluent channel (outer radius of 30 cm) with standpipe drains (Fig. 1). The mixing and effluent channels were divided up by solid walls into eight equally sized sections to receive and drain, respectively, water of different temperatures. Each effluent section had a separate drain back to the same reservoir from which water was pumped into that section’s associated mixing channel, to minimize the heating and chilling requirements (Fig. 1). The outer and inner swimming channel walls of each of the eight sections had forty 5 mm holes distributed along four offset rows over the height of the water column to reduce thermal stratification (Reiser et al. 2013). The swimming channel was 10 cm wide with a water depth of 10 cm, and a mid-channel circumference of 220 cm. The swimming channel was unobstructed except for thin (0.8 mm) radial dividers of clear polycarbonate between the eight sections that extended into the top 1 cm of the water column. These dividers did not interfere with the fish movements around the swimming channel but helped reduce surface flows between sections and shearing forces that would lead to thermal stratification (Chen et al. 2008; Reiser et al. 2013).

To create a temperature gradient ranging from 24 to 34 °C, water in each of five separate seawater reservoirs (45 L) below the preference chamber was individually thermostatted. The water was continuously aerated, and temperature regulated using submersible heaters or by recirculating water through stainless steel coils immersed in a chilled water bath. In all, a total of 1,600 W of heaters and two ¼ hp water chillers were required to create the temperature gradient. Water from the reservoirs was delivered by eight pumps (Eheim, Universal 600) into the eight mixing channels through ball-valves to regulate flow (5.06 ± 0.14 L/min per section). Dye tracking tests confirmed that most of the water pumped into each mixing channel section flowed directly across the swimming section and into the adjacent effluent channel to drain back to the originating reservoir. Oxygen saturation was tested in each of the eight zones in the swimming channel and ranged from 98.4 to 101.6%. To allow equilibration of water height across the reservoirs in case of slight discrepancies in the net flows in and out, each reservoir was connected to adjacent reservoir(s) of the closest temperature through a 1.3 cm diameter pipe at the bottom.

Mounted 162 cm directly above the swimming section, a ring of eight equally-spaced, white LED lights (2 W each), and four infrared lights (3 W each) were installed to provide uniform illumination. During the day (06:30–18:30), the white LED lights illuminated the chamber, while at night (18:30–06:30), an overhead, 1.5 W blue LED light indirectly lit the swim channel to provide minimal light for fish orientation, and the infrared lights enabled video monitoring. A USB camera (1 megapixel, 3.6 mm lens; ELP USB100W05MT-DL36) centered overhead (191 cm) continuously recorded fish position. The study apparatus was surrounded by a black curtain that excluded outside light and disturbances. For assigning temperatures to fish position, the swimming channel was divided into eight primary temperature sections (‘A’ through ‘H’) that were further divided into three subsections each resulting in a total of 24 temperature zones (Fig. 1; Reiser et al. 2013). Temperatures in the eight primary sections were continuously recorded to the computer (every 8 s) from digital temperature probes (DS18b20, ± 0.5 °C accuracy, ± 0.0625 °C resolution) attached to the middle of each section’s inner wall at mid-depth (Fig. 1) and could be viewed remotely to verify that temperatures remained stable throughout the experiment (Fig. S3). If the temperature in any zone deviated more than 0.5 °C for over 10 min, that data was excluded, or the trial was discarded due to temperature instability.

Prior to the start of an experiment, the system was filled with seawater and the temperature throughout was adjusted to match the acclimation temperature of the subject. One *Z. flavescens* was then released into a randomly selected zone in the swim channel. The fish had one hour of acclimation to the apparatus with no temperature gradient and then had one hour with positions recorded at the acclimation temperature before the temperature gradient was established (i.e., ‘no gradient’ measurement). Then the temperature was gradually changed in each reservoir until the desired temperature gradient was reached in the temperature preference chamber (over 1 to 1.5 h). After the temperature gradient stabilized, the 24 h temperature preference observations began, at 13:00 for the 27  °C treatments and 13:30 for the 31  °C treatments. To ensure no bias in preference for locations in the swimming channel, the temperature gradient was flipped 180° after half of the fish were tested from each acclimation group. After each trial, the fish was returned to the isolation basket in the acclimation tank and two replicates of temperatures were taken with a handheld digital thermometer, (CDN, DTW450) on the outer wall, mid-channel, and inner wall of the swim channel at three depths (1.0 cm, 5.5 cm, 10 cm). The average temperatures of the three depths at the three locations (i.e., nine temperatures total per subsection) were used to assign a temperature to each of the 24 subsections (A1, A2,…, H3) for each experiment. Between trials the system was disinfected with bleach and rinsed with freshwater.

### Aerobic scope data and statistical analysis

Oxygen consumption rates were calculated as the slope of the linear regression of the oxygen content decline as a function of time during the closed measurement period (Roche et al. 2013; Svendsen et al. 2016). The decline in oxygen content was evaluated for sufficient linearity using a minimum r-square threshold of 0.95. The oxygen consumption calculation was:

$${\dot {M}_{{\text{O2}}}}=\delta \left[ {{{\text{O}}_{\text{2}}}} \right] \times (\it{V_{{\text{chamber}}}}-\it{V_{\text{f}}})$$

where *Ṁ*_O2_ is the oxygen consumption rate (mg O_2_ h^−1^), delta O_2_ was the rate of change in oxygen content during intermittent respirometry, *V*_*chamber*_ was the volume of the respirometer, *V*_*f*_ was the fish’s volume (Ern et al. 2016; Svedson et al. 2016). The *Ṁ*_O2_ measurements were corrected by subtracting the background respiration, assuming a linear change in background *Ṁ*_O2_ over time. The standard metabolic rate (SMR) was found by creating a frequency histogram of the overnight *Ṁ*_O2_ values and fitting a double-normal distribution curve to separate periods of rest from periods of spontaneous activity. Standard metabolic rate was the mean of the lower distribution (Chabot et al. 2016; Steffensen et al. 1994). Maximum metabolic rate was the highest recorded *Ṁ*_O2_ value during a full 5 min measurement period at the maximum swimming speed (Roche et al. 2013).

To correct for mass-scaling effects, SMR and MMR were standardized to a common mass of 20 g using the following equation:

$${\dot {M}_{O2,20}}={\dot {M}_{O2}}\times{\left( {\frac{{20}}{{{\text{Mass}}}}} \right)^b}$$

Where *Ṁ*_O2,20_ is the mass-adjusted metabolic rate, *Ṁ*_O2_ is the non-adjusted metabolic rate, and Mass is the fish’s mass in grams. The scaling exponents (*b*) were determined empirically from the relationship between the log of measured SMR and log of body mass (0.73) and between the log of measured MMR and log of body mass (0.81, ANCOVA). The resulting mass-adjusted metabolic rates are referred to as SMR_20_ and MMR_20_. The mass-adjusted absolute aerobic scope (AS_20_) for each fish was calculated as the difference between SMR_20_ and MMR_20_ (Halsey et al. 2018):

$${\text{A}}{{\text{S}}_{{\text{2}}0}}\,=\,{\text{MM}}{{\text{R}}_{{\text{2}}0}}-{\text{SM}}{{\text{R}}_{{\text{2}}0}}$$

The data were assessed for normality using Shapiro-Wilk’s test and homogenous variance by conducting Levene’s test in RStudio (R Development Core Team 2010). F-tests were conducted to compare the variances of SMR_20_, MMR_20_, and AS_20_ values between the two acclimation temperature groups, and followed by the appropriate t-test to compare the groups.

### Swimming performance data analysis

Videos taken during each ten-minute cycle were used to find the critical swimming speed (*U*_crit_), as well as the two gait change speeds from pectoral fin to body-caudal fin swimming (*U*_p−c_) and from steady body-caudal fin swimming to burst and coast swimming (*U*_b−c_). The critical swimming speed was calculated following Brett (1964):

$${U_{{\text{crit}}}}=U\,+\,{U_i}\times\left( {\frac{t}{t_i}} \right)$$

where *U* was the speed before final swimming speed, *U*_*i*_ was the swimming speed increment (BL s^−1^), *t* was the length of time at the final swimming speed and *t*_*i*_ was the time interval at each speed. The *U*_p−c_ was calculated using the same equation, but *U* was the previous speed that the fish completed three full cycles of purely pectoral fin swimming before using body-caudal fin swimming for more than 5 s, and *t* is the time using pectoral fin swimming at the speed where the gait change occurred (Johansen and Jones 2011). Similarly, *U*_b−c_ was calculated from the same equation with speeds relating to that gait transition. The data were assessed for normality and then a t-Test assuming equal variances was used to compare *U*_p−c_, *U*_b−c_, and *U*_crit_ between the two acclimation treatments.

### Temperature preference data and statistical analysis

Videos from the overhead camera were analyzed using idTracker software (https://www.idtracker.es, Pérez-Escudero et al. 2014) to automatically generate X and Y coordinates for the fish’s location within the temperature preference chamber based on the assigned tracking parameters. The fish position was identified from a video frame every 20 s to avoid autocorrelation of position measurements, because the fish were able to swim around the entire swimming channel in less than 10 s (Behrens et al. 2012). The X and Y coordinates were then correlated to the locations of the 24 temperature zones of the swimming channel and each position was assigned an average temperature recorded in that zone. During the ‘no gradient’ observations (i.e., uniform temperature), the positions were assigned to the eventual temperatures of the zones after the gradient was established to determine if there was a selection bias not related to the temperature gradient.

To determine if the fish showed a temperature preference as opposed to random movement through available temperatures, the distribution of selected temperature values each hour of the 24 h preference test was compared with both the distribution of available temperatures and the distribution of observed positions during the ‘no gradient’ period. The available temperature distribution was based on 180 values equally distributed among all 24 temperature zones, to match the number of observations in one hour. ‘Non-choosing’ hours (Andreassen 2019; Behrens et al. 2012), when the fish did not show a temperature preference, were determined using quantile regression (SPSS v.28, IBM). If the first quartile, median, and third quartile values during the hour were all not significantly different (*p* ≥ 0.05) from either the available temperature distribution or the ‘no gradient’ observations for that fish, then that specific hour was removed from further analysis because the fish was a ‘non-chooser’ during that hour (Andreassen 2019). Removal of these data prevented biasing the T_pref_ towards the median of available temperatures if the fish were not selecting locations within the chamber.

The results of the temperature preference experiments were analyzed using two different methods. The first compared preference medians, and first and third quartiles, using a marginal linear model. The second used compositional analysis to compare the usage of the different temperature zones relative to availability within the swimming channel. The T_pref_ during all daytime or nighttime hours (excluding twilight hours when the lighting changed) was calculated as the median of temperatures selected, along with the first and third quartile, which represent the lower and upper thermal preference zone (Magnuson et al. 1979). A marginal linear model (MLM), using the linear mixed model procedures in SPSS (West et al. 2014), was used to test for the effect of acclimation temperature on the preference median, first and third quartiles. In addition, this analysis tested for differences among the available temperature gradient and three time periods: the ‘no gradient’ control observations, and the day and night temperature preferences. The marginal model used the time period (which included all available temperatures as one of the periods) and acclimation temperature as fixed factors and time period as repeated measures with subjects as fish identity. The interaction term of time period × acclimation temperature was not significant and, therefore, removed from the model. The chosen covariance structure was diagonal, based on model comparisons to find the lowest value of Hurvich and Tsai’s Criterion (AIC_c_). Multiple comparison p-values were corrected using the Holm-Bonferroni sequential correction (Gaetano 2018; Holm 1979).

The second analysis of temperature preference used compositional analysis of habitat use to determine non-random use among the temperature zones (Aebischer et al. 1993; Schram et al. 2013). Compositional analysis was used to discern the usage of temperatures compared with their availability within the preference chamber and to account for non-independence of proportional habitat use data (Aebischer et al. 1993). To conduct compositional analysis with the available sample size, data for the entire 24 h observation period were used, and the temperatures were combined into five bin ranges as the available ‘habitats’ (24–26, 26–28, 28–30, 30–32, and 32–34 °C). The relative temperature bin usage, or the preference ratio, was the proportion used over the proportion available (i.e., ‘usage’/‘availability’) to account for an unequal distribution of available temperatures within the preference chamber (Schram et al. 2013). The ‘usage’ frequency was the number of observations of fish position that fell within the temperature bin divided by the total number of observations, and ‘availability’ frequency was the number of temperature zones within each bin divided by the total number of temperature zones. A preference ratio of greater than 1.0 indicates the fish had a higher proportion of usage compared with the proportion available and a value below 1.0 means the fish used the temperature range less than its availability. To present the overall preference ratios in the figures, the geometric mean was used because of the log-normal distribution of probability ratios. To determine if there was non-random usage of available habitat, the ‘usage’ frequencies for ‘no gradient’ and during the 24 h preference observations were compared with the ‘available’ temperature frequencies using the ‘compana’ command in the adehabitatHS package (version 0.315; Calenge 2006) for RStudio (version 1.2.1335). If usage was determined to be non-random, this analysis then ranked temperature bins from least to most preferred, and determined which ranks were significantly different (Aebischer et al. 1993).

Multivariate Analysis of Variance (MANOVA) was conducted in RStudio to determine if there was a significant difference in preference between the two acclimation temperatures. To remove the unit-sum constraint on the compositional data (Aebischer et al. 1993) the preference ratios were replaced with the log ratio Q, calculated as the natural logarithm of the ‘usage’ frequency of the temperature bin divided by the ‘usage’ frequency at the middle temperature bin (i.e., 28–30  °C) and then subtracting the natural log of the ratio of the ‘availability’ frequency of that bin to the ‘availability’ frequency of the middle temperature bin (Schram et al. 2013).

$${\text{Q}} = \ln\left( \frac{{\text{usage}}^{{\text{frequency bin}}(i)}}{{\text{usage}}^{{\text{frequency at middle temp. bin (28--30}}\,^\circ{\text{C}})}} -\frac{{\text{availability}}^{{\text{frequency bin}}(i)}}{{\text{availability}}^{{\text{frequency at middle temp. bin (28--30}}\,^\circ{\text{C}})}}\right)$$

The values for Q were calculated for each fish in the five temperature bins (24–26, 26–28, 28–30, 30–32, and 32–34  °C) and were used in the MANOVA analysis to compare the two acclimation groups. All statistical tests used a significance level of α = 0.05.

## Results

### Aerobic scope

**Fig. 2:**
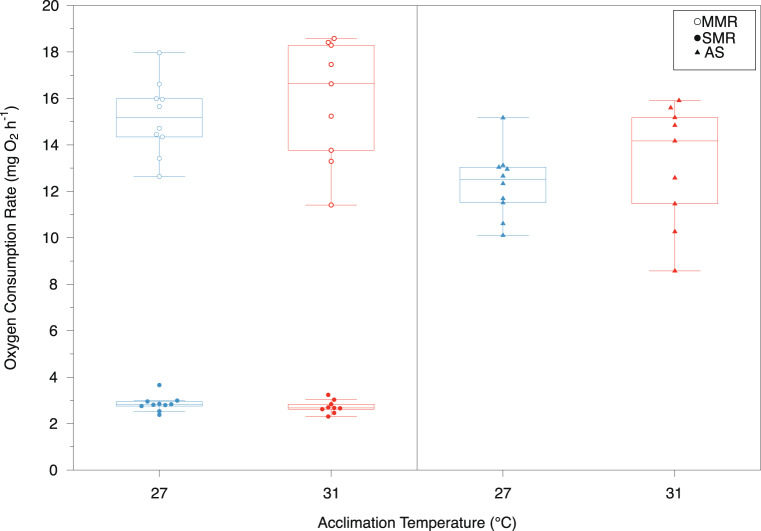
Mass-adjusted standard metabolic rate (SMR_20_; closed circles), maximum metabolic rate (MMR_20_; open circles), and aerobic scope (AS_20_ = MMR_20_–SMR_20_; triangles) of *Zebrasoma flavescens* in a swimming respirometer at two acclimation temperatures 27  °C (*n* = 10; blue) and 31  °C (*n* = 9; red). Metabolic rates were adjusted to a common mass of 20 g

Although mean fish mass was not significantly different between the two acclimation groups (27 °C, 23.2 ± 5.4 g; 31 °C, 19.6 ± 4.8 g; *t*(17) = 1.54, *p* = 0.14), mass was found to have a significant effect on metabolic rate. The scaling exponent for standard metabolic rate (SMR) was not significantly different between acclimation temperatures (ANCOVA, homogeneity of slopes, *F*(1,15) = 1.14, *p* = 0.30); therefore, common scaling exponents were used to normalize metabolic rates to a common mass of 20 g. For SMR the scaling exponent was 0.73 (*F*(1,16) = 43.0, *p* < 0.001, *r*^2^ = 0.77), and for maximum metabolic rate (MMR) was 0.81 (*F*(1,16) = 32.1, *p* < 0.001, *r*^2^ = 0.68). After correcting for mass differences, standard metabolic rates, maximal metabolic rates and aerobic scope were similar in both acclimation groups (Fig. 2). The SMR_20_ was not significantly different in *Z. flavescens* acclimated to 27–31 °C (*t*(17) = 0.95, *p* = 0.36; 2.86 ± 0.34 mg O_2_ h^−1^ and 2.72 ± 0.28 mg O_2_ h^−1^, respectively, mean ± *SD*). The MMR_20_ in both acclimation groups was five times higher than SMR_20_, although more variable. As with SMR_20_, acclimation to warmer waters had no significant effect on MMR_20_ (*t*(17) = −0.76, *p* = 0.45), although the 27  °C treatment group had a slightly lower MMR_20_ on average, 15.16 ± 1.60 mg O_2_ h^−1^, compared with the 31  °C treatment group, at 15.90 ± 2.59 mg O_2_ h^−1^ (Fig. 2). Consequently, absolute aerobic scope (AS_20_), or the difference between MMR_20_ and SMR_20_, had a similar range in fish from the two acclimation temperatures (Fig. 2). Acclimation to warmer water had no significant effect on AS (*t*(17) = −0.93, *p* = 0.37; 12.30 ± 1.46 mg O_2_ h^−1^ at 27℃ and 13.18 ± 2.59 mg O_2_ h^−1^ at 31  °C). Overall, following acclimation to the warmer temperature, the metabolic rates and aerobic capacity of *Z. flavescens* remained largely the same.

### Swimming performance

**Fig. 3:**
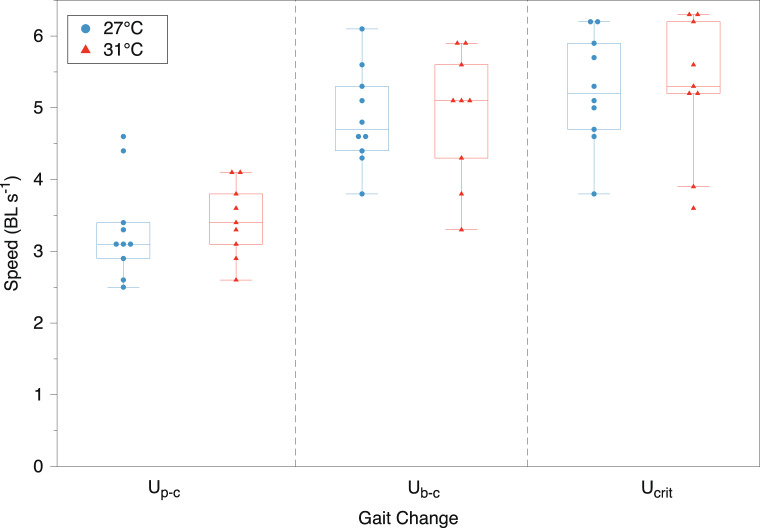
Swimming performance measures for *Zebrasoma flavescens* acclimated to 27  °C (*n* = 10; blue) and 31  °C (*n* = 9; red). The gait transition *U*_p−c_ (circles) was the speed at which the fish changed from pectoral fin to body-caudal fin swimming, *U*_b−c_ (triangles) was the speed at which the fish changed from steady body-caudal swimming to unsteady burst-and-coast swimming, and *U*_crit_ (squares) was the critical swimming speed before exhaustion

There was a wide range of swimming performance measures among individual fish, although the acclimation temperature had no significant effect on *Z. flavescens* swimming performance (Fig. 3). The gait transition speed from pectoral to caudal fin swimming (*U*_p−c_) averaged 3.3 ± 0.7 BL s^−1^ and 3.4 ± 0.5 BL s^−1^ at acclimation temperatures 27 and 31  °C, respectively, which were not significantly different (*t*(17) = −0.44, *p* = 0.67). However, the *Z. flavescens* were capable of prolonged swimming beyond this gait transition. The transition from body-caudal fin swimming to the cyclical burst and coast swimming (*U*_b−c_) occurred at mean values of 4.8 ± 0.7 and 4.9 ± 0.9 BL s^−1^ for *Z. flavescens* acclimated to 27 and 31  °C, respectively, (*t*(17) = −0.12, *p* = 0.90). The *U*_b−c_ transition occurred just below the critical swimming speeds (*U*_crit_): 5.3 ± 0.7 BL s^−1^ at 27  °C and 5.3 ± 1.0 BL s^−1^ at 31  °C (*t*(17) = −0.04, *p* = 0.90; Fig. 3).

### Temperature preference: fish behavior

**Fig. 4:**
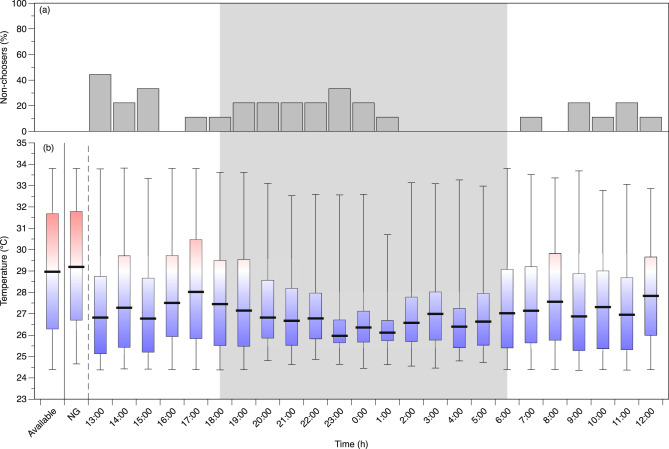
Temperature preferences of *Zebrasoma flavescens* acclimated to 27  °C (*n* = 9) over the 24 h observation period. **a** The percentage of individuals identified as ‘non-choosers’ (showing no temperature selection) at each hour that were not used to calculate temperature preference. **b** Average median (horizontal bar), first and third quartiles (box), and temperature ranges (whiskers) chosen during each hour of all fish that showed a temperature preference. ‘Available’ is the average available temperatures in the preference chamber and ‘no gradient’ (NG) is the average of observed use of the eventual temperature zones during the hour of observations when there was no temperature gradient (uniform acclimation temperature, 27  °C). The shaded gray region demarcates nighttime.

**Fig. 5:**
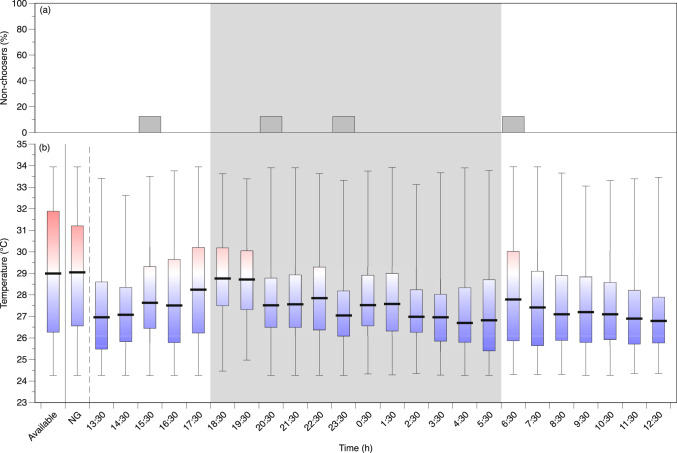
Temperature preferences of *Zebrasoma flavescens* acclimated to 31  °C (*n* = 8) over the 24 h observation period. **a** The percentage of individuals identified as ‘non-choosers’ (showing no temperature selection) at each hour that were not used to calculate temperature preference. **b** Average median (bar), first and third quartiles (box), and temperature ranges (whiskers) chosen during each hour of all fish that showed a temperature preference. ‘Available’ is the average available temperatures in the preference chamber and ‘no gradient’ (NG) is the average of observed use of the eventual temperature zones during the hour of observations when there was no temperature gradient (uniform acclimation temperature, 31  °C). The shaded gray region demarcates nighttime.

Throughout the 24 h period of observation in the temperature gradient, most *Z. flavescens* continued to explore all areas of the swimming channel (Figs. 4 and 5). While some would settle in a narrow range of temperatures for a period of time, other times the fish would continually swim around the entire chamber, resulting a wide range of temperatures experienced each hour. The temperature distributions each hour were compared with the available temperature distribution to distinguish if the fish showed a temperature preference as opposed to random use of the temperature gradient. In addition, each hour was compared with the distribution of positions during the ‘no gradient’ period to determine if there was a position bias unrelated to the temperature gradient. If the distributions were not different from these two controls, then the fish was identified as ‘non-choosing’ during that hour of the test. Less than half of the fish were ‘non-choosers’ at any given hour, and these were not included in temperature preference calculations to avoid biasing the temperature preference towards the median of available temperatures (Figs. 4a and 5a).

The first hour of the 24 h preference experiment following establishment of the temperature gradient had the highest percentage of non-choosers for fish acclimated to 27  °C (Fig. 4a). About 67% (*n* = 6) of *Z. flavescens* acclimated to 27  °C were ‘non-choosers’ for at least one of the 24 hours compared with only 37.5% (*n* = 3) of *Z. flavescens* acclimated to 31  °C. The 31 °C fish also had fewer hours during the preference test were fish displayed ‘non-choosing’ behavior (Fig. 5a). All fish, however, showed a temperature preference during most of the 24 h observation period, and the average preferred temperatures (i.e., medians) during each hour in both acclimation groups tended to be lower than the median of available temperatures (Figs. 4b and 5b). For *Z. flavescens* acclimated to 27  °C, the average T_pref_ tended to decrease in the middle of the night (i.e., 23:00–1:00) with a narrower interquartile range (IQR) than during the daytime hours (Fig. 4b). The *Z. flavescens* acclimated to 31 °C had similar average preferred temperatures during the day and at night with wider IQRs at night compared with fish acclimated to 27  °C (Figs. 4 and 5).

### Temperature preference: median and quartiles analysis

Both acclimation groups had a similar T_pref_ (medians) close to 27 °C, with a preference zone from the first quartile (Q1) to the third quartile (Q3) of approximately 25.7 °C to 29 °C. No significant differences were found between acclimation temperatures across all time periods in median, Q1, or Q3 (MLM; *F*(1, 15.3) = 2.03, *p* = 0.18; *F*(1, 16.5) = 0.004, *p* = 0.95; *F*(1, 21.2) = 0.24, *p* = 0.63, respectively; Table 1). The time period, however, had a significant effect on all three quartiles (MLM, median: *F*(3, 16.1) = 26.2, *p* < 0.001; Q1: *F*(3, 18.0) = 3.73, *p* = 0.03; and Q3: *F*(3, 17.9) = 29.54, *p* < 0.001).

**Table 1:** The first quartile (Q1), median, and third quartile (Q3) temperatures (mean ± S.D.) from the available temperatures in the preference chamber (ranging from 24 to 34 °C), and *Zebrasoma flavescens* usage during the ‘no gradient’, daytime and nighttime periods during the 24 h preference trial for both acclimation temperatures (27 °C, *n* = 9; and 31 °C, *n* = 8). During ‘no gradient’ usage, temperatures were uniform at the respective acclimation temperature, and values represent the eventual temperatures at the fish positions after the gradient had been established

	Temperatures (°C)								
	27 °C Acclimation					31 °C Acclimation			
Period	Q1		Median	Q3		Q1	Median	Q3	
Available Temperatures	26.3 ± 0.2	29.0 ± 0.1^a^		31.5 ± 0.3^a^		26.3 ± 0.1	29.0 ± 0.1 ^a^	31.5 ± 0.2^a^	
‘No Gradient’ Usage	26.7 ± 1.1	29.3 ± 0.8^a^		31.8 ± 0.5^a^		26.6 ± 1.1	29.0 ± 1.4 ^a^	31.2 ± 1.1^a^	
Day Preference	25.7 ± 1.1	27.4 ± 1.5^b^		29.5 ± 2.1^b^		25.7 ± 1.4	27.1 ± 1.6 ^b^	29.0 ± 1.8^b^	
Night Preference	25.4 ± 0.8	26.3 ± 0.9^b^		28.3 ± 1.2^b^		26.1 ± 1.2	27.2 ± 1.5 ^b^	29.0 ± 1.6^b^	

Different letters represent significant differences within each quartile (*p* < 0.05). No significant differences between acclimation groups were found

Post-hoc t-tests with Holm-Bonferroni correction found that the two control periods, (1) based on the distribution of available temperatures and (2) based on location usage during the ‘no gradient’ period, were not statistically different from each other, indicating that the fish were not biased to a specific location while the temperature was homogenous in the preference chamber (Table 1). There were also no significant differences between day and night temperatures for all three quartiles during exposure to the gradient. However, both day and night temperature preference medians were significantly lower than the available temperature or ‘no-gradient’ usage (*p* < 0.001), as were Q3 temperatures (*p* < 0.001; Table 1). Although a main effect of time period was found for Q1 temperatures, and day and night Q1 values averaged 0.2 to 1.3 °C lower than the available and ‘no gradient’ values, the post-hoc tests found no significant differences. The results indicate that for both acclimation groups, the fish’s preferred temperatures (median) and upper range of the preference zone (Q3) were similarly lower during both day and night compared with the distribution of available temperatures (Table 1).

### Temperature preference: compositional analysis of habitat use

**Fig. 6:**
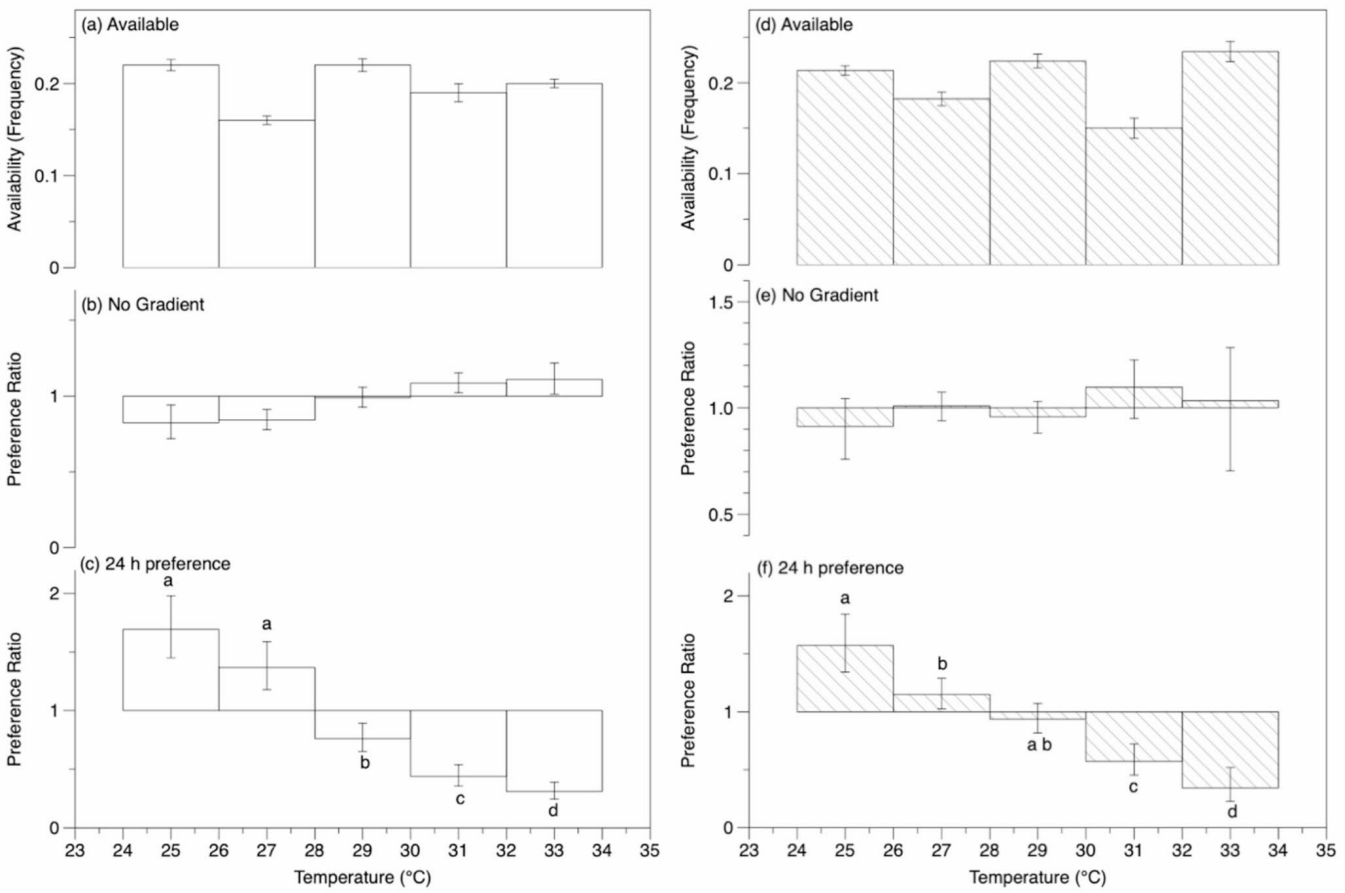
Temperature preference of *Zebrasoma flavescens* acclimated to 27 °C (**a**–**c**; *n* = 9) or 31 °C (**d**–**f**; *n* = 8; shaded). **a**, **d** The average proportion (frequency) of available temperatures in the temperature preference chamber. **b**, **e** Geometric means of the preference ratio (proportion used/proportion available) in each eventual temperature zone before the temperature gradient was establish and the water in the preference chamber was a uniform 27–31 °C. **c**, **f** Geometric means of the preference ratio during 24 h observations in the temperature gradient excluding twilight hours. Error bars are S.E.M. Different letters denote significant differences among temperature bins based on rank compositional analysis, *p* < 0.05.

The zone usage during the ‘no gradient’ hour was random for both the 27 °C acclimation group (lambda = 0.435, *p* = 0.31, Fig. 6b) and the 31 °C acclimation group (lambda = 0.144, *p* = 0.09, Fig. 6e) indicating there was no preference for any locations prior to establishing the temperature gradient. During the 24 h observation period in the gradient, however, *Z. flavescens* acclimated to 27 °C had a significant non-random use of temperature ranges (lambda = 0.145, *p* = 0.016; Fig. 6c) with the highest preference for the coldest temperature range. Based on results of a non-parametric rank-based comparison of zone usage (Aebischer et al. 1993), the two coldest temperature ranges, 24–26 °C and 26–28 °C were not statistically different from each other but they were significantly more preferred than the warmer temperatures, from 28 to 34 °C. The warmest temperature areas (32–34 °C, followed by 30–32 °C) were the least preferred (use was less than the availability). The fish acclimated to 31 °C also had significant non-random use of the temperature gradient during the 24 h observation (lambda = 0.0733, *p* = 0.016; Fig. 6f). Based on ranks of habitat usage, the preference for the coldest temperatures, 24–26 °C, was significantly greater than 26–28 °C but not from 28 to 30 °C. As observed for the 27 °C acclimation group, the least preference was shown for the warmest temperatures (Fig. 6f). The usage of temperature ranges was not significantly different between the two acclimation groups (MANOVA, *F*(1, 15) = 1.347, *p* = 0.31).

## Discussion

Climate change has led to rising sea surface temperatures along with more frequent and severe marine heatwaves in tropical coral reef ecosystems (Hoegh-Guldberg et al. 2014). It is predicted that these changes may result in shifts in latitudinal distributions of species (Dahms and Killen 2023; Perry et al. 2005) and affect the fitness of coral reef fishes (Johansen and Jones 2011; Jutfelt 2020). Previous studies have shown, however, that even among closely related species and genera, physiological and behavioral responses to elevated temperatures are highly variable and species specific (Feary et al. 2014; Johansen and Jones 2011; Nilsson et al. 2009).

We assessed how acclimation to an elevated temperature affected the physiology and behavior of *Zebrasoma flavescens*, an important herbivore that supports coral reef health in the Hawaiian Archipelago. Our findings contribute to understanding species resilience amidst climate-driven temperature changes in marine ecosystems. Fish acclimated to elevated temperatures for at least four weeks (31 °C, or 4 °C above the present summer monthly maximum) had similar aerobic metabolic performance as those held at 27 °C, in aerobic scope and in both standard and maximum metabolic rates (Fig. 2). Consistent with metabolic measures, indicators of swimming performance were also similar in both acclimation groups (Fig. 3). These results suggest that for these measures of fitness *Z. flavescens* are able to completely compensate for long-term elevated ocean temperatures, at least up to 31 °C. For the behavioral response, both acclimation groups tended to select cooler waters at, or below, the current maximum monthly average of 27 °C (Table 1; Figs. 4 and 5). Therefore, despite metabolic and swimming performance compensations to increased temperatures, temperature preference remained the same, and towards the mid to low end of this species’ normal habitat temperature range.

### Aerobic scope

The ‘oxygen- and capacity-limited thermal tolerance’ (OCLTT) hypothesis links the effects of temperature on performance (i.e., aerobic scope) as a mismatch between a decreased oxygen supply to the tissues and increased oxygen demand at elevated temperatures (Lefevre et al. 2021; Pörtner 2010; Pörtner and Knust 2007). As ectotherms, the metabolic rates of fishes are expected to increase exponentially with acute increases in temperature, reflected in a higher SMR. This response is usually moderated with acclimation due to compensatory changes that can occur over days to weeks and return rate processes towards the pre-exposure level (Seebacher et al. 2014). These compensatory adjustments can occur at many biological levels, including biochemical, cellular, morphological and physiological (Johansen et al. 2021; Jutfelt 2024; Schulte 2015). The acclimation response may only be partial compensation, effectively reducing the response to temperature change, or, if returning to the same levels, complete compensation to maintain constant rates over a range of temperatures (Precht 1958; Seebacher et al. 2014). The MMR also tends to increase with temperature, but may peak and then decrease, or plateau, at higher temperatures as cardiorespiratory capacity becomes limited. The differential response between SMR and MMR results in a temperature where aerobic scope (AS) is maximized and decreases above or below this optimal temperature (T_opt_; Ern et al. 2016; Messmer et al. 2017; Pörtner 2010; Pörtner and Knust 2007; Rummer et al. 2014). Under the OCLTT hypothesis, fitness should be maximal at T_opt_ as the fish has the greatest capacity to support metabolic functions. Contrary to our expectations, we found that acclimation to the higher temperature in *Z. flavescens* did not significantly change AS, despite this temperature (31 °C) falling above the current habitat range. It may be that AS is not reduced until even higher temperatures in this species, or the optimum lies somewhere between our two acclimation temperatures.

Responses to temperature increases appear to be very species-specific, and some species maintain their AS over a wide range of temperatures (Lefevre et al. 2021). For example, although the overall response of 10 tropical damselfishes (Family Pomacentridae) to an increase from 29 °C to 32 °C was a reduction in AS and swimming performance, individual variation was evident, as some fish showed no significant change while others were strongly affected (Johansen and Jones 2011). As with *Z. flavescens*, other fishes have shown complete compensation of metabolic rates with thermal acclimation to elevated temperatures (Johansen et al. 2021; Sandblom et al. 2014). Previous studies have provided evidence that both supports the OCLTT hypothesis (Pörtner 2001, 2021; Pörtner et al. 2017; Schulte 2015) and disputes it (Jutfelt et al. 2021; Kirby et al. 2020; Norin et al. 2014; Raby et al. 2016). This lack of consistency has led to the conclusion that this hypothesis cannot be universally applied (Lefevre et al. 2021) and our results support this. *Zebrasoma flavescens* did not show a reduction in physiological oxygen supply at higher temperatures that would limit physiological functions, and therefore does not explain the species’ current upper habitat temperatures.

### Swimming performance

Swimming performance is paramount for coral reef fish survival, affecting multiple aspects of their daily existence, such as, escaping predators, navigating currents, and foraging (Fulton et al. 2005; Plaut 2001). For the first time, we assessed swimming performance in *Z. flavescens*, which like many other surgeonfishes (Family Acanthuridae), primarily use the pectoral-fins during routine swimming (Fulton 2007). The effect of elevated temperatures on swimming performance are variable among species with some experiencing a significant performance decrease because of a reduction in AS (Johansen and Jones 2011; Johansen et al. 2014; Sandblom et al. 2014). Maximum swimming performance in fishes is often assumed to occur at temperatures where aerobic scope is also maximal (Kelsch and Neill 1990) and as with AS in the present study, *Z. flavescens*’ swimming performance was unaffected by acclimation temperature, indicating complete compensation for the effects of temperature (Fig. 3). As swimming speed increases, pectoral fin swimmers switch to the use of body and caudal fin undulations, and this gait transition speed (*U*_p−c_) occurred in *Z. flavescens* between 2.5 and 4.5 BL/s, within the range of values (1.4 − 4.8 BL/s) reported for other acanthurids (Fulton et al. 2005). This gait transition involves a switch from powering swimming with the pectoral musculature to the axial muscles (Drucker and Jensen 1996). The *Z. flavescens* were still capable of prolonged swimming (30 min periods) above this speed using steady body-caudal fin undulations, suggesting that this gait is still primarily supported by aerobic metabolism (Svendsen et al. 2010). Once a switch to unsteady burst-and-coast swimming (*U*_b−c_) occurred, fatigue followed shortly thereafter, with MMRs measured just prior to this point (Fig. 3). Critical swimming speeds (*U*_crit_) were as high as 6.3 BL s^−1^, and again similar to those found for other acanthurids (Fulton 2007).

### Temperature preference

Temperature preference (T_pref_) has been studied in annular preference chambers, as in the present study, or using a shuttlebox apparatus consisting of two connected chambers, one warmer than the other, where the temperature is adjusted according to the fish’s movement between chambers (Behrens et al. 2012; Christensen et al. 2021; Myrick et al. 2004). The shuttlebox system can test over a broader temperature range whereas the annular preference chambers limit the range of temperatures available but have no hiding spots and allows the test subject to move freely in a temperature gradient without the need to learn how to regulate temperature by moving between chambers (Behrens et al. 2012; Myrick et al. 2004; Reynolds and Casterlin 1980).

We used two methods of data analysis to determine temperature preferences: one using the median and interquartile range (IQR) of selected temperatures to allow comparison to previous studies, and another that can correct for potential biases in the availability of thermal ranges in an annular preference chamber. In both methods, we recommend excluding periods when fish do not show any preference or are moving essentially at random and exploring all areas within the chamber equally (i.e., “non-choosing”; Andreassen 2019; Behrens et al. 2012; Christensen et al. 2021). Individual differences in fish behaviors, or personalities, and non-choosing behavior of some fish has been reported previously in annular preference chambers (Andreassen 2019; Behrens et al. 2012). Most temperature preference studies have used the median to report thermal preference (Behrens et al. 2012; Cocherell et al. 2014; Gräns et al. 2010; Magnuson et al. 1979; Reiser et al. 2013). The thermal range (i.e., the interquartile range from the first to third quartiles) is included because fish do not select a single temperature but instead select or move within a temperature range (Magnuson et al. 1979; Grans et al. 2010). The Z. *flavescens*, regardless of acclimation temperature, had a median preference of around 27 °C, with a range (Q1 to Q3) of around 25.7 °C to 29 °C (Table 1), within the available gradient of 24 to 34 °C. This median preference is similar to the highest monthly average sea surface temperature (SST) in summer around O’ahu.

A potential problem with annular chambers is that the unequal distribution of available temperatures could bias the results (Fig. 6a and d). This issue can be ameliorated by adjusting the temperatures in each section to try to make different temperature sections equally available (McMahon et al. 2020). Alternatively, the data can be analyzed to take this inequality into account, using techniques from tracking of free-ranging animals to examine relative habitat use, as was done by Schram et al. (2013; Aebischer et al. 1993). Compositional analysis of habitat use corrects for temperature availability by determining the proportion used compared with the proportion available. However, in the compositional analysis, we were restricted to comparing the preference for broader temperature ranges (i.e., 2 °C bins) because the sample size (i.e., number of individuals tracked) limits the number of ‘habitats’ that can be examined in the analysis (Aebischer et al. 1993).

Based on ranks of habitat usage, *Z. flavescens* from both acclimation temperatures (27 and 31 °C) showed the greatest preference for the coldest temperature range, 24–26 °C, followed by 26–28 °C, which are within the annual SST fluctuations around Hawaiʻi (Fig. 6c and f; Kappel et al. 2017; Wedding et al. 2018). Compositional analysis of habitat use suggested lower preferred temperatures (< 26 °C) than when using the median (~ 27 °C). It may be that the median is biased upwards when the preferred temperature is at the low end of the range available, because higher temperature areas are more available when the fish explores the swimming channel. Also, because the lowest temperature range appeared to have the highest preferences (Fig. 6c and f), we cannot discount the possibility that the fish may have preferred even cooler temperatures, had they been available. A previous study on temperature preference of *Z. flavescens* using a shuttlebox system found a mean preference that varied from 23 °C during the day down to 19 °C at night (overall mean of 21 °C; Reynolds and Casterlin 1980), which is surprising as this nighttime preference is well below the temperatures available in their natural habitat (> 23 °C). Additionally, both acclimation groups least preferred 32–34 °C followed by 30–32 °C, which are in the potential range of elevated SST or SST compounded with marine heatwaves (Fig. 6c and f; Hoegh-Guldberg et al. 2014).

Several other studies on T_pref_ of tropical fishes have found similar results, where regardless of acclimation temperature (current or end-of-century SST), fish preferred the same temperatures, close to current habitat temperatures. For example, five-lined cardinalfish (*Cheilodipterus quinquelineatus*) preferred their average summer SST (Nay et al. 2015), barramundi (*Lates calcarifer*) preferred a temperature within the current habitat range (Norin et al. 2014), and at acclimation temperatures from 25 to 31 °C green chromis (*Chromis viridis)* maintained a preferred temperature close to the long-term summer average SST (28.9 °C; Habary et al. 2017). The elevated acclimation temperatures from our experiment and these prior studies did not alter the temperature preference of the various fish species tested, potentially indicating that temperature preference may not be phenotypically plastic in these species.

In fishes, temperature preference has been used as a proxy for the optimal temperature for fitness of physiological and biochemical processes, including swimming performance, growth and aerobic scope (Habary et al. 2017; Jobling 1981; Kelsch and Neill 1990; Macnaughton et al. 2018; Magnuson et al. 1979). Accordingly, we would expect that as body temperature moves further away from the preferred temperature, measures of fitness should decline. We did not find that to be the case in *Z. flavescens* acclimated to 31 °C. While T_pref_ remained close to 27 °C or lower, AS and swimming performance were not reduced at 31 °C. The match between temperature preference and fitness measures has been found to vary with species. Some studies have found a correlation, with maximum AS or growth occurring at the preferred temperatures, for example, in *C. viridis* (Habary et al. 2017) and yellowtail kingfish (*Seriola lalandi*; Larios-Soriano et al. 2021). Others have found a mismatch, with preference occurring at temperatures below the optimal AS, such as in *L. calcarifer* (Norin et al. 2014) and in individual zebrafish (*Danio rerio*; Ripley et al. 2022). Martin and Huey (2008) argue that because of the rapid decrease in fitness at higher temperatures, ectotherms should actually choose temperatures that are below the optimal temperature to maximize fitness over time. In addition, the multiple performances multiple optima (MPMO) hypothesis states that the optimal temperatures for fitness measures and performance may not all occur at the same temperature (Clark et al. 2013). For example, the spiny chromis (*Acanthochromis polyacanthus)* showed metabolic acclimation to maintain AS at temperatures 3 °C above current SST, but these temperatures also resulted a reduction in reproductive output and growth (Donelson et al. 2010, 2011). As a result, other areas of physiological performance in *Z. flavescens*, such as growth or reproduction, may be optimal at the temperature preference and be the driver for behavioral thermoregulation (Ripley et al. 2022).

### Ecological significance

Thermal niches and optimized aerobic performance are essential components to understand suitable habitats for coral reef species. The *Z. flavescens* are an economically and ecologically important herbivore in coral reef ecosystems (Walsh et al. 2004) and their response to increased temperatures may help predict population responses to future climate change (Hoey et al. 2016). Long-term temperature increases have led to changes in population abundance and distribution with shifts to higher latitudes in various tropical species, subsequently altering the community structure (Dahms and Killen 2023; Lefevre et al. 2021; Perry et al. 2005). While many tropical reef fishes are thought to be near their upper temperature limits and show decreases in AS and swimming performance with a 2–3 °C increase in temperature (Habary et al. 2017; Hoey et al. 2016; Johansen and Jones 2011; Rummer et al. 2014), Z. *flavescens* show the capacity to fully compensate with acclimation to a 4 °C increase. This response may be due to their more subtropical habitat, allowing greater resilience to predicted increases in SST (Bernardi et al. 2018). The ability to maintain performance at 31 °C suggests that the success of these fish in future warmer oceans will be influenced more by changes in ecological factors, such as habitat availability, food resources, and predator dynamics, rather than physiological constraints.

The complete compensation in metabolic rates (SMR and MMR), and therefore energy demands, would indicate that at higher SST *Z. flavescens* may not change their resource use from the environment. However, herbivorous fishes have been shown to increase in abundance following marine heatwaves, associated with the loss of corals and increased algal cover as a food resource (Hoey et al. 2016; Olsen et al. 2022). In addition, if *Z. flavescens* is able to adjust physiologically to maintain performance, they would be at a competitive advantage over coral reef species that are not able to completely compensate to the elevated temperatures (Sandblom et al. 2014). Despite maintaining the same AS at higher temperatures, T_pref_ was not altered, and *Z. flavescens* continued to behaviorally select cooler temperatures (generally below 29 °C and as low as 24–26 °C). If this temperature preference is reflective of the species optimal habitat or other aspects of fitness, it could result in a northward shift of the population along the Hawaiian archipelago following changes in suitable habitat with rising SST temperatures (Eble et al. 2011; Habary et al. 2017).

### Future studies

The present study examined responses to acclimation to an elevated, steady temperature. This study used a single tank for each acclimation temperature, and future studies should include replicate tanks to account for potential tank effects and improve the robustness of the findings. Given that AS and swimming performance did not change at these higher temperatures, future studies should examine higher and lower temperatures in order to generate a complete thermal performance curve for *Z. flavescens*, which may reveal a thermal optimum (Rummer et al. 2014). In addition, periodic heat waves (occurring over time periods that may be too short to allow acclimation processes to complete) may also be more extreme in the future, and it is unknown if acclimation to higher temperatures will protect fishes during heatwave exposure or if they will be closer to upper thermal limits (Tran and Johansen 2023).

The T_pref_ for *Z. flavescens* was at the low end of our experimental temperatures; therefore, future studies should include temperatures below 24  °C to determine if they prefer even colder temperatures. It also appears that preference temperature in this species does not match optimal AS and swimming performance, but it may correlate with other aspects of fitness, such as growth, digestion efficiency or reproductive potential. Further studies should examine these aspects in response to temperature change, which could also reveal if there is a trade-off for the maintained metabolic rates at higher temperatures (Donelson et al. 2010) or if the MPMO hypothesis applies to this species.

Our study assessed a single generation acclimated to 31 °C over multiple weeks, however, it has yet to be determined how exposure to elevated temperatures affects multiple generations (Jutfelt 2020). Generally, adaptation to elevated ocean temperatures would occur through natural selection and would result in preferred fitness traits that are suitable for the environmental conditions (Jutfelt 2020).

### Conclusion

We found that *Z. flavescens* displays a high degree of resilience to elevated temperatures, maintaining both aerobic scope and swimming performance at levels exceeding its typical habitat range. At least up to 31 °C, this response in *Z. flavescens* did not fit the OCLTT hypothesis of reduced oxygen delivery capacity and suggests a robust capacity to withstand predicted increases in SST. Temperature preference, however, was at the low end of the normal habitat range and was unchanged with acclimation, suggesting other aspects of fitness may account for this preference and be suboptimal at elevated temperatures. This metabolic stability may provide a competitive advantage in warming seas, although its preference for cooler temperatures may result in a future habitat shift. These findings provide a framework for assessing thermal resilience in other reef species, emphasizing the complex interplay of habitat suitability factors that will ultimately shape species responses to changing ocean temperatures (Jorgensen et al. 2012; Wilson et al. 2010).

## Supplementary Information

Below is the link to the electronic supplementary material.

Supplementary material 1 (DOCX 976.7 kb)

## Data Availability

The data supporting the findings of this study are available in Fig Share at 10.6084/m9.figshare.28963478.
