# Supplementary material for: Aerobic scope and temperature preference in yellow tang (Zebrasoma flavescens) at current and elevated sea temperatures
Source: J Comp Physiol B. 2025 Aug 26;195(5):535–53. doi: 10.1007/s00360-025-01627-y (PMC12602642; doi:10.1007/s00360-025-01627-y)
Supplement: Supplementary file 1 — Supplementary material 1 (DOCX 976.7 kb) [file 360_2025_1627_MOESM1_ESM.docx]

Supplementary Materials


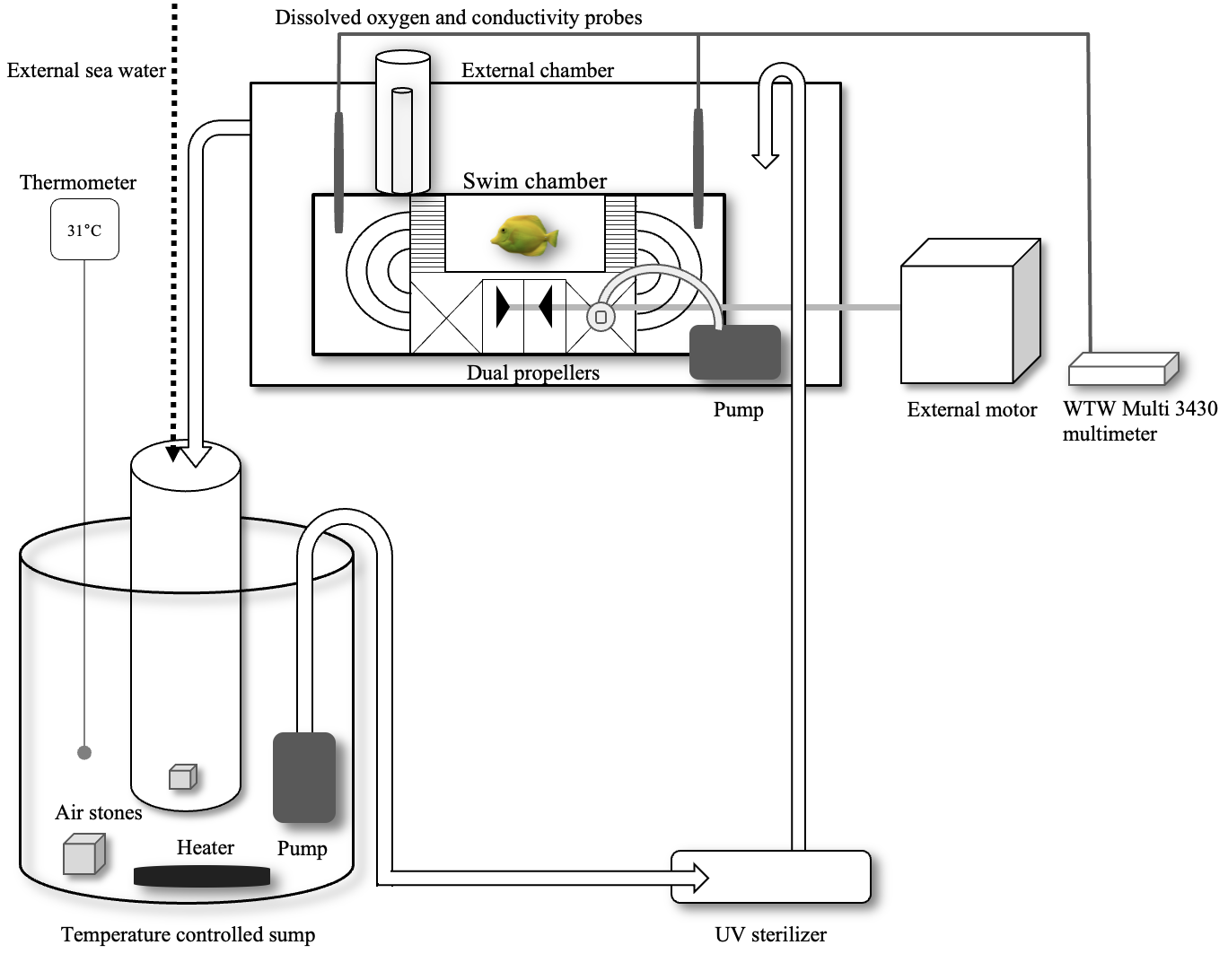


**Fig. S1** Intermittent flow respirometry system with an aerated, temperature-controlled sump (52.0 L) that. Water from the sump was pumped through a UV sterilizer to reduce microbial activity before entering the external water tank (52.4 L), which supplied water to the 6.7 L swim chamber (Schakmann et al. 2020). Conductivity and dissolved oxygen probes were placed in the swim chamber, with all measurements transmitted to a computer via the WTW 3430 multimeter


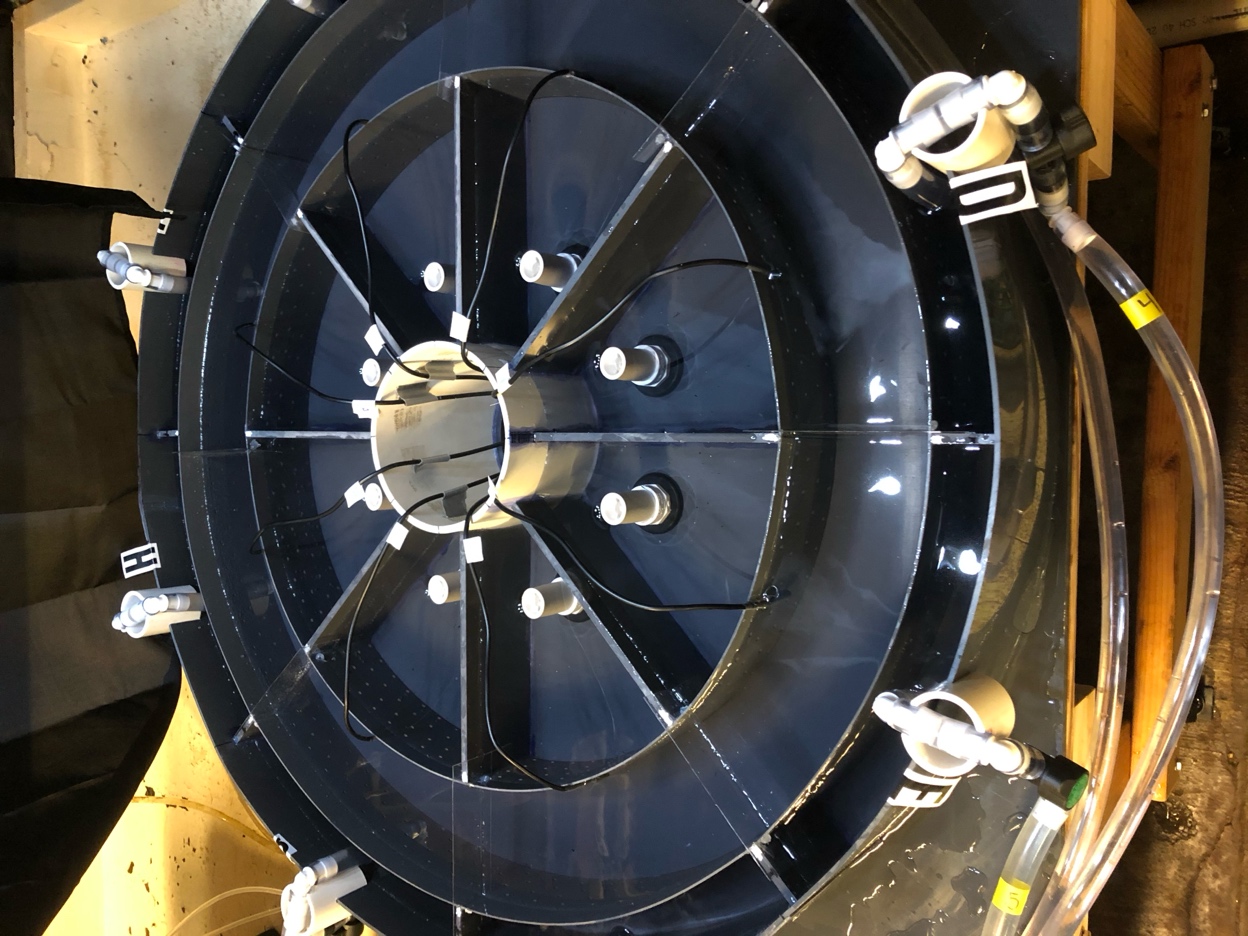


**Fig. S2** The annular temperature preference chamber (TPC) with a total outer radius of 45.0 cm. The TPC consisted of a 10 cm-wide swim channel where fish were placed, surrounded by an outer mixing channel (5 cm wide) that received temperature-regulated seawater pumped from reservoirs and an inner effluent channel (10 cm wide) with standpipe drains for water recirculation back to the reservoirs (Myrick et al. 2004; Schram et al. 2013). The swim channel had a mid-channel circumference of 220 cm and was divided into eight sections by thin (0.8 mm) radial dividers extending 1 cm into the water surface to minimize surface mixing. Water temperatures were continuously monitored using temperature sensors affixed to the middle of the inner wall of the swim channel in each temperature zone (e.g., at A2, B2, C2, … H2)


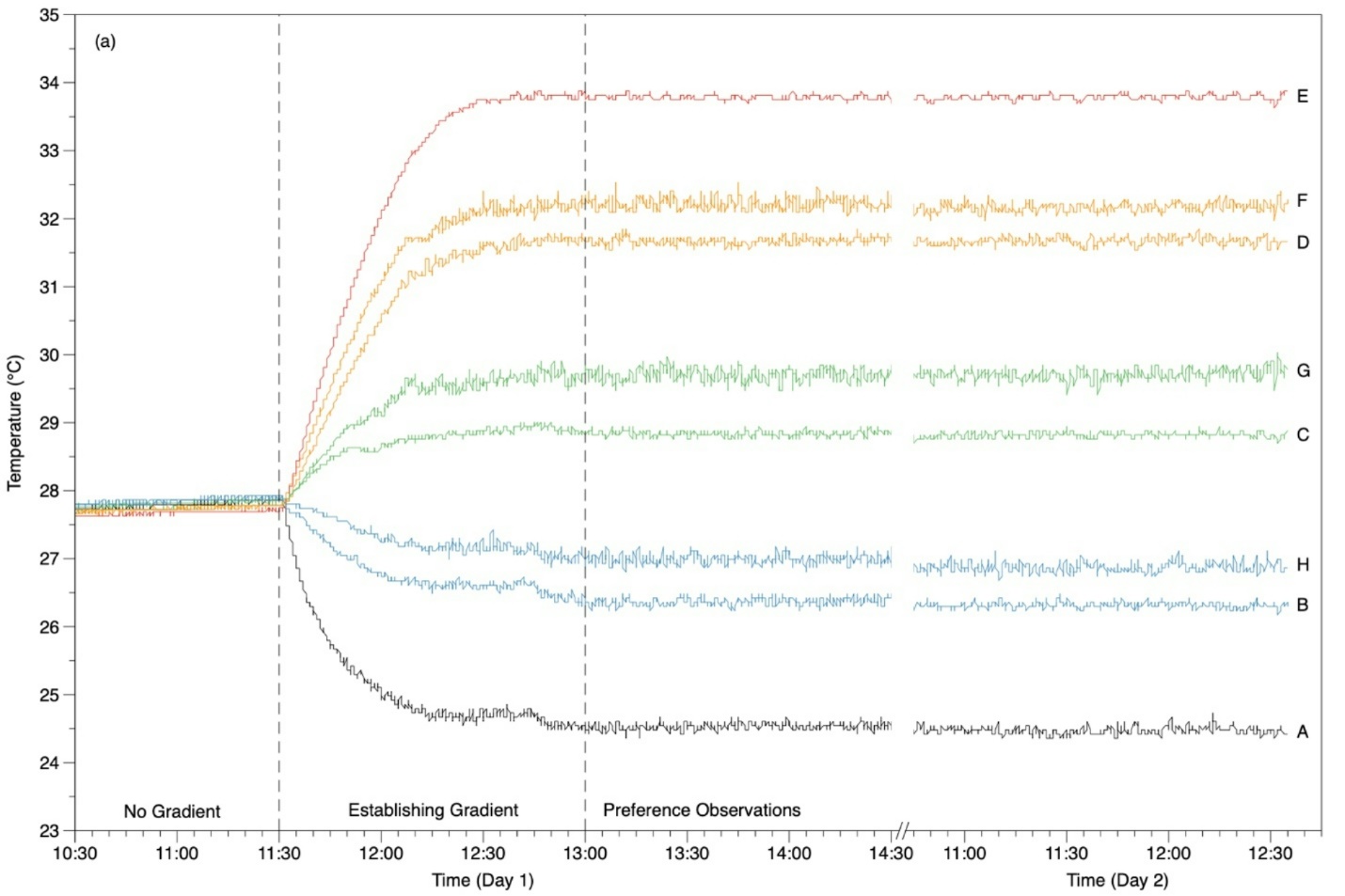


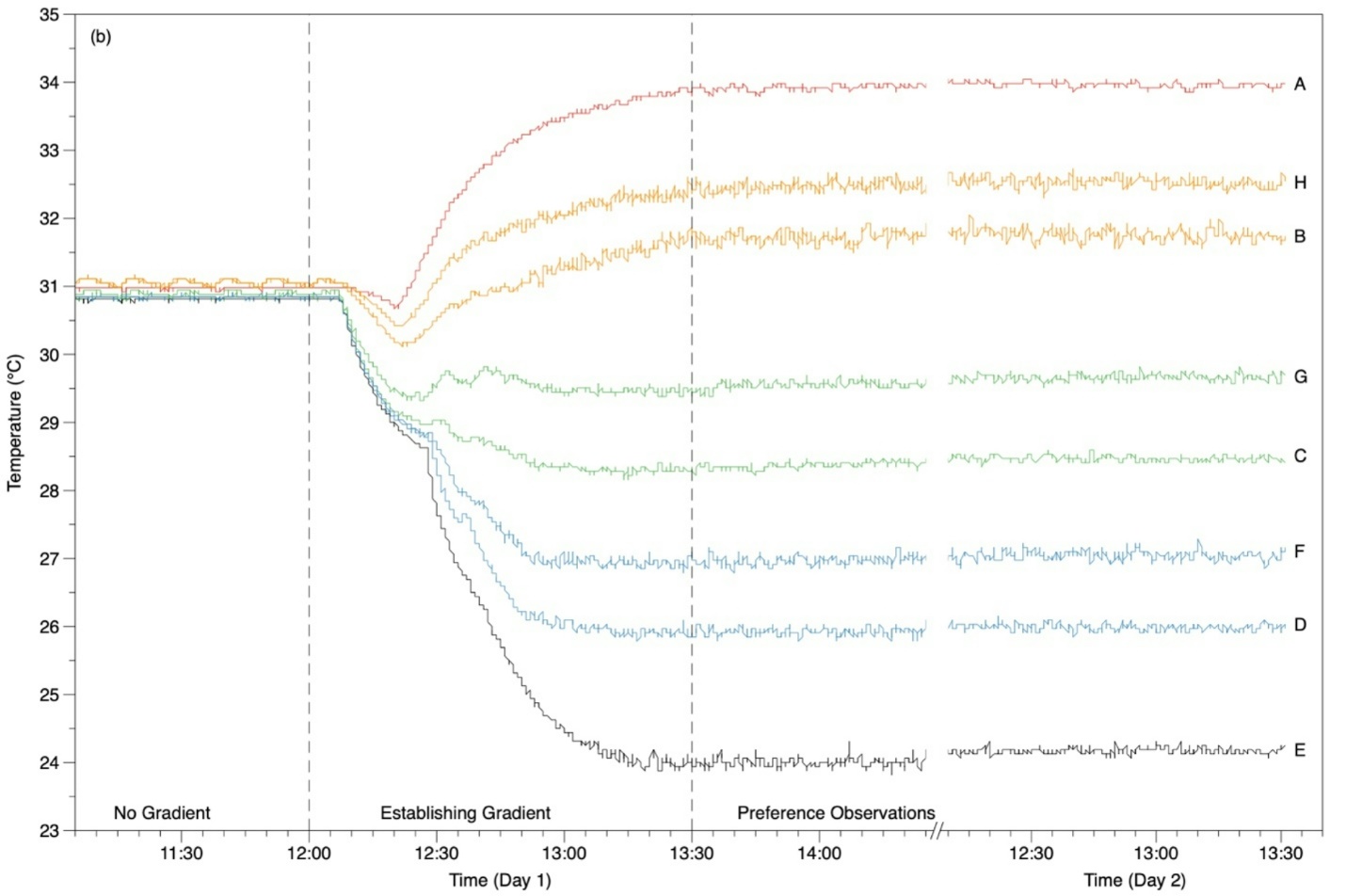


**Fig. S3** Water temperatures from the digital temperature sensors in the eight temperature zones (A-H) from an experiment with a fish acclimated to 27℃ (A) and 31℃ (B). Following one hour after introduction into the preference chamber, observations consisted of one hour with all zones at the acclimation temperature (i.e., ‘No Gradient’ 27℃ or 31℃), then a period when the temperature gradient was established, and finally 24 hours of temperature preference observations
